# Reduced risk of recurrent pneumothorax for sirolimus therapy after surgical pleural covering of entire lung in lymphangioleiomyomatosis

**DOI:** 10.1186/s13023-021-02081-z

**Published:** 2021-11-03

**Authors:** Teiko Sakurai, Toru Arai, Masaki Hirose, Kensuke Kojima, Tetsuki Sakamoto, Yoshinobu Matsuda, Chikatoshi Sugimoto, Hyung-Eun Yoon, Yoshikazu Inoue

**Affiliations:** 1 Department of General Thoracic Surgery, National Hospital Organization Kinki-Chuo Chest Medical Center, Nagasone-cho 1180, Sakai, Osaka 591-8555 Japan; 2 Clinical Research Center, National Hospital Organization Kinki-Chuo Chest Medical Center, Nagasone-cho 1180, Sakai, Osaka 591-8555 Japan; 3 Department of Psychosomatic Internal Medicine, National Hospital Organization Kinki-Chuo Chest Medical Center, Nagasone-cho 1180, Sakai, Osaka 591-8555 Japan

**Keywords:** Lymphangioleiomyomatosis, Pneumothorax, Sirolimus, Vascular endothelial growth factor D, Total pleural covering, Surgical pleural covering

## Abstract

**Background:**

Patients with lymphangioleiomyomatosis (LAM) frequently experience pneumothorax. Although sirolimus is the standard therapy for LAM, its effect on pneumothorax is controversial. Recently, total pleural covering (TPC) and modified TPC (mTPC) were introduced as surgical treatment options for pneumothorax for patients with LAM. However, the effect of sirolimus on the recurrence of pneumothorax in patients who underwent the treatments is still uncertain. We hypothesized that some clinical factors including sirolimus treatment could predict postoperative recurrence of pneumothorax. In order to clarify this hypothesis, we retrospectively analyzed the clinical data from 18 consecutive patients with LAM who underwent 24 surgical pleural covering of entire lung (SPC) as 17 TPC and 7 mTPC against pneumothoraces from surgical database between January 2005 and January 2019, and we determined the predictors of postoperative recurrence.

**Results:**

Of the 24 surgeries of SPC, 14 surgeries (58.3%) had a history of two or more ipsilateral pneumothoraces, and 11 surgeries (45.8%) had a history of ipsilateral pleural procedures before SPC. Sixteen surgeries (66.6%) in 12 patients received treatment of sirolimus after SPC (sirolimus group). With a median follow-up time of 69.0 months after SPC, four surgeries (16.6%) in three patients had a postoperative recurrence, and the 5-year recurrence-free survival (RFS) after SPC was 82.9%. In patients with postoperative recurrence, serum level of vascular endothelial growth factors D was significantly higher than that in those with non-recurrence (3260.5 vs. 892.7 pg/mL, *p* = 0.02), and the rate of sirolimus treatment in the recurrence group was significantly lower than that in the no-recurrence group (0 vs. 80%, *p* = 0.006). The log-rank test showed that the RFS of the sirolimus group (sirolimus use after SPC) was significantly better than that of the non-sirolimus group (*p* = 0.001), and no significant difference was observed for other factors.

**Conclusion:**

We first reported sirolimus might effectively suppress the recurrence of pneumothoraces in LAM patients who received SPC. Sirolimus induction after SPC (TPC or mTPC) might be a feasible option for frequent pneumothorax in LAM.

**Supplementary Information:**

The online version contains supplementary material available at 10.1186/s13023-021-02081-z.

## Background

Lymphangioleiomyomatosis (LAM) is a rare multi-organ disease that predominantly affects women [1]. There are two types of LAM: sporadic and as a manifestation of tuberous sclerosis complex (TSC). Progressive multiple pulmonary cysts result in respiratory failure, and the cysts might also cause frequent pneumothoraces [2]. Almoosa et al. reported that recurrence rates were 66% after conservative therapy, 27% after chemical pleurodesis, and 32% after surgery [3]. The official American Thoracic Society/Japanese Respiratory Society clinical practice guidelines recommend that patients with LAM be offered ipsilateral pleurodesis after initial pneumothorax, rather than waiting for recurrence, before intervention with a pleural symphysis procedure (conditional recommendation, very low confidence) [4]. However, physicians occasionally encounter cases with recurrence even after pleural procedures or cases without re-expansion persistently, which require additional managements.

Lung transplantation is the final therapeutic option for end-stage LAM patients. However, previous pleural procedures might be likely to have intra- or postoperative severe hemorrhage associated with lung transplantation due to pleural adhesion [3]. Recently, Kurihara et al. have reported a surgical technique called total pleural covering (TPC) to prevent both severe pleural adhesion and recurrent pneumothorax. In TPC, surgeons cover the entire visceral pleura with oxidized regenerated cellulose (ORC) mesh to reinforce it [5–7]. In addition, Noda et al. reported five cases successfully treated by TPC technique modified with a preceding coverage of air-leak points with polyglycolic acid (PGA) sheets (modified TPC, mTPC) [6].

After the mechanistic target of rapamycin (mTOR) inhibitor sirolimus was proved to stabilize lung function in LAM by clinical trials [8, 9], sirolimus has been used as standard therapy in LAM [4, 10]. Sirolimus was also found to reduce the size of lymphangioleiomyomas and chylous effusion [11]. Takia et al. and Zhoh et al. had reported that the frequency of pneumothorax decreased in number after sirolimus induction in a small number of populations [12, 13]. On the other hand, pneumothorax is reported to be an adverse event of sirolimus [9]. Therefore, the effect of sirolimus on pneumothorax still needs to be clarified.

Recently, some factors have been reported as signs of progressive disease in LAM. We reported that serum vascular endothelial growth factor D (VEGF-D) level was a useful diagnostic biomarker for LAM [10, 14]. In addition, serum VEGF-D levels correlate with pulmonary dysfunction and reflect the effect of sirolimus treatment [15–17]. Postmenopausal status and low pulmonary function were associated with disease progression of LAM [18].

We hypothesized that certain clinical factors including sirolimus treatment would predict postoperative recurrence of pneumothorax in LAM. In order to clarify the hypothesis, we retrospectively analyzed the clinical data of our consecutive LAM patients who underwent surgical pleural covering of entire lung (SPC) as TPC or mTPC for pneumothorax from our surgical database and investigated outcome and possible predictors for postoperative recurrence of pneumothorax.

## Methods

### Patient cohort

**Fig. 1:**
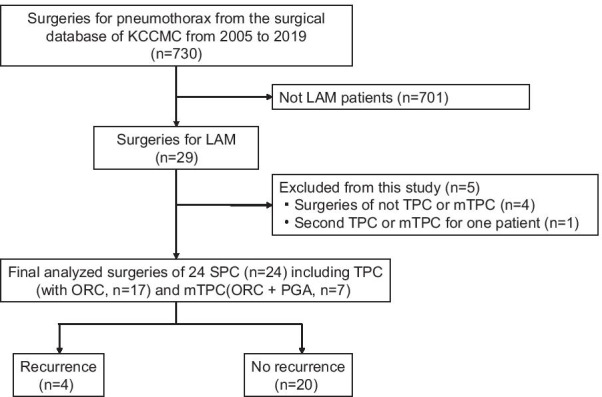
Flow diagram of the study population. Twenty-four surgical pleural covering of entire lung including 17 total pleural covering and 7 modified total pleural covering from 18 patients from the consecutive database of surgeries for pneumothorax (n = 730). Abbreviation: LAM, lymphangioleiomyomatosis; SPC, surgical pleural covering; TPC, total lung covering;,mTPC, modified total pleural covering; ORC, oxidized regenerated cellulose; PGA, polyglycolic acid

Study populations who underwent TPC or mTPC as SPC cases were identified using the surgical database of the National Hospital Organization Kinki-Chuo Chest Medical Center (KCCMC) between January 2005 and January 2019, a total of 730 surgeries for pneumothorax were performed at KCCMC. Of these, 24 surgeries in 18 patients with LAM, wherein SPC was performed were finally analyzed in this study (Fig. 1). In patients with bilateral SPC, the data for each side were analyzed. Non-SPC cases were excluded, and if a patient underwent multiple SPC ipsilaterally, only the initial SPC was included for analysis and the rest were excluded (five surgeries were excluded as follows; two non-SPC in two patients: single wedge resections, two non-SPC: overlapping SPC due to relapse, one surgery: second SPC).

KCCMC has been a designated International LAM Clinic by the LAM Foundation (Cincinnati, OH, USA) and the diagnosis of LAM in KCCMC was based on international guidelines [4, 10]. In this study, a definite histological diagnosis of LAM was obtained from all patients, in which LAM cells were confirmed by immunohistochemistrical staining of smooth muscle actin and HMB45 using the lung specimens obtained by transbronchial or surgical lung biopsy [19].

### Clinical parameters

From the medical records in KCCMC, we collected the following data on the clinical characteristics and treatment details during the perioperative period of SPC: age, status of menopause, sex, body mass index (BMI), comorbidity associated with LAM, serum albumin level, history of pneumothorax or pleural procedures (including those performed in other institutions), bronchodilator use, oxygen therapy requirement, surgical approach, and postoperative complications. We also collected the following data during the follow-up period: all episodes of pneumothorax, pleural procedure, the treatment period and amount of sirolimus, transplantation, and date and cause of death. All episodes of pneumothorax, which occurred during the visit of KCCMC, were radiologically confirmed by computed tomography (CT) or plain chest film. The episodes treated in other hospitals were counted on the basis of a careful patients’ interview.

Serum VEGF-D level was measured during the perioperative period by using a commercially available enzyme-linked immunosorbent assay kit (R&D Systems Inc., Minneapolis, MN, USA) in our laboratory, Clinical Research Center, KCCMC [17].

To quantify low lung attenuation that was associated with cystic changes in the lung, all quantitative high-resolution CT examinations were performed using a 16 multi-detector CT scanner (HiSpeed Ultra 16; GE Healthcare, Chicago, IL, USA). To evaluate the low attenuation area (LAA), the percentage of low attenuation volume (%LAV) was calculated as lung volume in areas with less than − 950 Hounsfield units at full inspiration [20, 21].

### Sirolimus administration and the definition of the “sirolimus group” and “non-sirolimus group”

Sirolimus was administered once a day with a starting dose of 1–2 mg/day, and the dose was regulated according to its efficacy, adverse events, and blood levels. In KCCMC, the patients paused sirolimus whenever they experienced pneumothorax, at least until pneumothorax was resolved.

To evaluate the relationship between the administration of sirolimus and postoperative relapse, we divided the surgeries according to the status of sirolimus administration after SPC into two groups: a “sirolimus group” that initiated or continued sirolimus over a month from initial SPC to ipsilateral recurrence or last follow-up and a “non-sirolimus group” that did not take sirolimus until postoperative recurrence after SPC or last follow-up. The surgery with sirolimus initiation after postoperative recurrence was classified as the “non-sirolimus group”. There was no patient who had sirolimus beforehand and withdrawn after the first pneumothorax and/or SPC (Such patients were planned to classify to “non-sirolimus group”).

We compared the patients’ characteristics between the patients with and without recurrence of pneumothorax, where appropriate. We compared factors between patients with and without sirolimus in the same manner.

### Surgical pleural covering of entire lung for pneumothorax

**Fig. 2:**
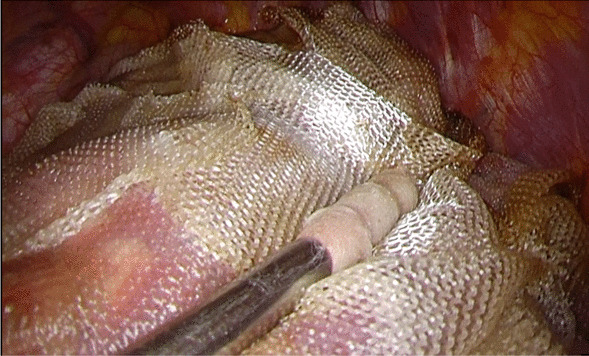
Total pleural covering by video-assisted thoracoscopic surgery. The surgeon covers the entire visceral pleura with the oxidized regenerated cellulose mesh without a gap. This white mesh turns acidic and is absorbed from the thoracic cavity

All surgical procedures were performed under general anesthesia. Video-assisted thoracoscopic surgery (VATS) is defined as surgery with a maximum incision length of 8 cm [22]; surgery with an incision length over 8 cm is classified as thoracotomy. After surgery, all patients had 20F or 24F chest tubes connected to the drainage system, set from -10 cmH_2_O to -5 cmH_2_O negative pressure. In this study, as SPC, we covered the entire visceral pleura with reinforcing materials, such as an ORC mesh (Surgicel®, Johnson & Johnson, Brunswick, NJ, USA) according to the procedure of Kurihara et al. (TPC) [5]. We also added from one to two sheet of PGA sheet (Neovail®, GUNZE, Tokyo, Japan) for a preceding coverage of air-leak points which was decided from necessity of the surgeon according to the procedure of Noda et al. (mTPC) [6]. Kurihara et al. recommended more than 10 ORC meshs for TPC. In our TPC, from 8 to 11 sheets of ORC mesh were required to cover entire lung. In addition to ORC mesh, we used one or two sheets of PGA sheet which were added for mTPC. The image of TPC by VATS is shown in Fig. 2. Before tube removal, the patient was asked to cough, and if a bubble was observed in the water column of the drainage system, air leakage was proved to exist. The number of days from the SPC until the disappearance of air leakage was counted from the medical records to determine the presence of persistent air leakage (PAL). For the evaluation of postoperative complications, adverse events within 30 days after SPC were included by using the Clavien-Dingo classification [23]. An additional table file shows this in more detail (see Additional file 1). Postoperative PAL was defined as air leakage prolonged for at least 5 days [24].

### Statistical analysis

The primary endpoint of the outcome was recurrence-free survival (RFS) from pneumothorax after the initial SPC. RFS was computed from the time when SPC was done to the event of interest or last follow-up until June 30, 2020. Recurrence of pneumothorax was defined as the first event of ipsilateral pneumothorax after SPC.

The descriptive statics for patients’ characteristics were analyzed using the Wilcoxon rank-sum test or Fisher’s exact test, where appropriate. RFS was estimated using the Kaplan–Meier method and survival curves were compared using the log-rank test. For comparison in the log-rank test, the continuous variables were divided into two groups by a median value. We evaluated the recurrence predictive value of clinical parameters, including age, BMI, comorbidity associated with LAM, serum VEGF-D level, %LAV, natural menopause, episodes of pneumothorax before SPC, oxygen use after SPC, bronchodilator use, and sirolimus use.

All statistical analyses were two-tailed, and a significance level of 0.05 was used. All statistical analyses were performed with EZR version 1.52 (Saitama Medical Center, Jichi Medical University, Saitama, Japan), which is a graphical user interface for R version 4.0.2 (The R Foundation for Statistical Computing, Vienna, Austria). More precisely, it is a modified version of R commander version 2.7-0, designed to add statistical functions frequently used in biostatistics [25].

## Results

### Patients’ characteristics

This study included 18 patients with LAM who had 24 surgeries (SPC including 17 TPC and 7 mTPC). The median follow-up time from the patients’ first visit to KCCMC was 80.5 months (range 51.5–125.4 months). The median age at which SPC was performed was 37.0 years (range 35.0–40.7 years), with a median postoperative follow-up time of 69.0 months (range 34.2–109.3 months) after SPC. Two patients terminated follow-up in KCCMC because one was referred to another institution, and one died from respiratory failure.

The clinical characteristics of patients treated with SPC according to the presence or absence of postoperative recurrence are presented in Table 1. This study included only one surgery in one patient with TSC-LAM and ten surgeries in eight patients with angiomyolipoma (AML) located in the kidney and liver in terms of comorbidities associated with LAM. When SPC was performed, all patients were yet to achieve natural menopause, though two patients had undergone a uterine resection and bilateral ovary removal due to endometrial carcinoma or uterine fibrosis, and two patients took a gonadotropin-releasing hormone (GnRH) agonist. The median incidence of ipsilateral pneumothorax before SPC was 2.0 (range 1.0–2.2), and 14 surgeries (58.3%) accompanied two or more events of ipsilateral pneumothorax. Eleven surgeries (45.8%) in 11 patients had a history of ipsilateral pleural procedures (five pleurodesis and eight surgical procedures) before SPC. Ten patients (55.5%) had a history of bilateral pneumothorax, and six of these had undergone SPC bilaterally in KCCMC. Overall, 16 surgeries (66.6%) in 12 patients who used sirolimus after SPC were included in the sirolimus group. However, two surgeries in one patient who started sirolimus after a bilateral recurrence were included in the non-sirolimus group.

**Table 1:** Clinical characteristics of surgical pleural covering of entire lung (total pleural covering and modified total pleural covering) in patients with LAM perioperatively per the presence of postoperative recurrence

Characteristics	Entire cohort (n = 24)	No recurrence (n = 20)	Recurrence (n = 4)	*P* value
Age, year^a^	37.0 (35.0–40.7)	37.0 (35.0–41.0)	39.5 (35.7–40.7)	0.72
Female, sex	24 (100)	20 (100)	4 (100)	–
Body mass index^a^	20.1 (18.6–21.1)	20.3 (18.8–21.2)	17.4 (15.5–19.7)	0.14
Comorbidities^a^				
TSC	1 (4.1)	0 (0)	1 (25)	0.16
AML	10 (41.6)	8 (40)	2 (50)	1.0
Serum albumin^a^, g/dL	4.2 (4.0–4.5)	4.2 (4.0–4.5)	4.2 (4.0–4.4)	1.0
Serum VEGF-D^a^, pg/mL	938.1 (715.4–1324.8)	892.7 (662.6–1091.2)	3260.5(1416.7–3718.1)	0.02
%LAV^a^, %	11.4 (4.4–20.0)	11.1 (4.2–20.0)	12.5 (9.8–16.4)	0.56
Pneumothorax episode^a^, times	2 (1–2.2)	2.0 (1–2.2)	1.5 (1–3.2)	0.96
History of surgical procedure^a^	8 (33.3)	7(35)	1(25)	1.0
History of pleurodesis^a^	5 (20.8)	4(20)	1(25)	1.0
Oxygen therapy				
Before SPC	1 (4.1)	1 (5)	0	1.0
After SPC	4 (16.7)	3 (15)	1 (25)	0.54
Bronchodilator treatment^a^	3 (12.5)	2 (10)	1 (25)	0.43
Sirolimus treatment^b^	16 (66.6)	16 (80)	0(0)	0.006
Approach				
VATS	20 (83.3)	17 (85)	3 (75)	0.54
Thoracotomy	4 (16.7)	3 (15)	1 (25)	
Postoperative complication				
PAL (≥ 5 days)	9 (37.5)	7 (35)	2 (50)	0.61
Death	1 (4.1)	0	1 (25)	0.16

Median (interquartile range), number (%)

AML, angiomyolipoma; LAM, lymphangioleiomyomatosis; LAV, low attenuation volume; PAL, persistent air leakage; SPC, surgical pleural covering of entire lung; SLB, surgical lung biopsy. TSC, tuberous sclerosis complex; VATS, video-assisted thoracoscopic surgery; VEGF-D, vascular endothelial growth factor D

^a^Preoperative data

^b^Postoperative data

As the surgical approach, 20 surgeries (83.3%) were VATS, and the rest were thoracotomy. During the follow-up period in KCCMC, one patient died of respiratory failure, and none underwent lung transplantation.

### Outcome after surgical pleural covering of entire lung

**Fig. 3:**
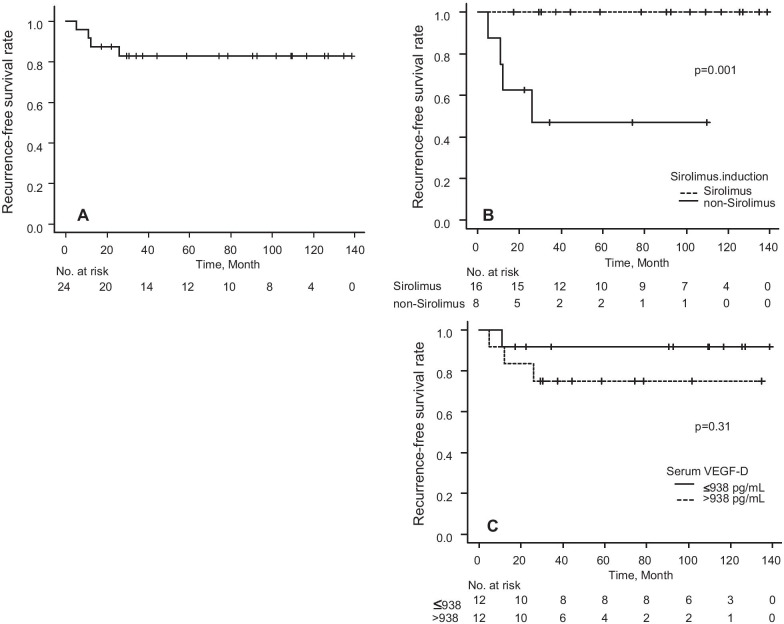
Kaplan–Meier curves of recurrence-free survival (RFS) after surgical pleural covering including total pleural covering and modified total pleural covering for pneumothorax in lymphangioleiomyomatosis. **A** Kaplan–Meier curve of RFS in total surgeries (n = 24). Five years RFS is 82.9%. **B** Kaplan–Meier curves of RFS in the sirolimus (n = 16) and the non-sirolimus groups (n = 8). The RFS in the sirolimus group is significantly better than that in the non-sirolimus group (*p* = 0.001, log-rank test revealed). **C** Kaplan–Meier curves of RFS in the high serum vascular endothelial growth factors D (VEGF-D) group (> 938 pg/mL, n = 12) and in the low serum VEGF-D group (≤ 938 pg/mL, n = 12). There are no significant differences between the high serum VEGF-D level and the low VEGF-D group (*p* = 0.31)

Overall, four surgeries (16.6%) in three patients had postoperative recurrent pneumothorax with a median postoperative time of 11.4 months (range 9.4–15.4 months). There was a 1-year recurrence-free rate of 87.5% and a 5-year recurrence-free rate of 82.9% in the study cohort (Fig. 3A). In the four surgeries with recurrence, three surgeries were associated with one episode of relapse, and the another was associated with two episodes during the postoperative follow-up period.

The postoperative complications within 30 days of SPC included PAL in 9 surgeries (one surgery, grade I; seven surgeries, grade IIIa; one surgery, grade IIIb), according to the Clavien-Dingo classification.

### Predictors of postoperative recurrence

The serum VEGF-D level in the recurrence group was significantly higher than that in the no recurrence group (3,260.5 pg/mL vs. 892.7 pg/mL, *p* = 0.02) and 80 percent of the patients in the no recurrence group were treated with sirolimus, and 0% of those in the recurrence group were treated with sirolimus (*p* = 0.006; Table 1). Therefore, to clarify that the factors resulting in the progression or severity of LAM might also predict postoperative recurrence, the factors enlisted as the predictors of postoperative recurrence were evaluated using the log-rank test, except for menopausal status.

RFS in the sirolimus group was significantly superior to that in the non-sirolimus group (*p* = 0.001; Fig. 3B). Moreover, all surgeries in the sirolimus group were without postoperative recurrence. Conversely, there was no significant difference between the two groups divided by the median value of serum VEGF-D levels (Fig. 3C) (*p* = 0.31). In addition, no significant differences were observed between age, BMI, the existence of AML, %LAV suggesting LAA, episodes of pneumothorax before SPC, oxygen use, and bronchodilator use. An additional figure file shows this in more detail (see Additional file 2).

### Details of sirolimus administration

The difference between the clinical characteristics of the sirolimus (n = 16) and non-sirolimus groups (n = 8) is shown in Table 2. No significant difference was observed. In the sirolimus group that included 16 surgeries in 12 patients, sirolimus was initiated in 15 surgeries and restarted in one surgery after a median postoperative time of 34.8 months (range 8.6–52.0 months); six surgeries (37.5%) were associated with sirolimus initiation or resumption within a year of SPC. Overall, the median administration time of sirolimus during follow-up in KCCMC was 26.8 months (range 18.1–73.1 months) in the sirolimus group. An additional table file shows more detailed data (see Additional file 3).

**Table 2:** The difference in characteristics by the presence of sirolimus use after surgical pleural covering of entire lung (total pleural covering and modified total pleural covering)

Variable	Sirolimus (n = 16)	Non-sirolimus (n = 8)	*P* value
Age^a^, year	37.0 (35.0–38.5)	39.5 (34.2–43.2)	0.55
Body mass index^a^	20.4 (19.2–21.5)	18.9 (17.6–20.0)	0.08
Comorbidities^a^			
TSC-LAM	0	1 (12.5)	0.33
AML	6 (37.5)	4 (50)	0.67
Serum albumin^a^, g/dL	4.3 (4.3–4.8)	4.0 (3.9–4.4)	0.17
Serum VEGF-D^a^, pg/mL	910 (655–1182)	938 (907–3078)	0.28
%LAV^a^, %	11.7 (3.6–20.9)	9.7 (6.4–12.0)	0.87
Pneumothorax episode^a^, times	2 (1–2.7)	2 (1–2.2)	0.89
History of surgical procedure^a^	6 (37.5)	2 (25)	0.66
History of pleurodesis^a^	4 (25)	1 (12.5)	0.63
Oxygen therapy^b^	3 (18.8)	1 (12.5)	1.0
Sirolimus induction before SPC	1 (6.2)	0	1.0
Bronchodilator treatment^a^	2 (12.5)	1 (12.5)	1.0
Approach,			
VATS	13 (81.2)	7 (87.5)	1.0
Thoracotomy	3 (18.8)	1 (12.5)	
Postoperative complication			
PAL (≥ 5 days)	6 (37.5)	3 (37.5)	1.0

Median (interquartile range) number(%)

AML, angiomyolipoma; LAV, low attenuation volume; PAL, persistent air leak; TSC, tuberous sclerosis complex; SPC, surgical pleural covering entire lung; VATS, video-assisted thoracoscopic surgery; VEGF-D, vascular endothelial growth factors D

^a^Preoperative data

^b^Postoperative data

The dose of sirolimus in the last follow-up at KCCMC was as follows: 3 mg (n = 1), 2 mg (n = 6), and 1 mg or less (n = 5). Though one patient experienced the first pneumothorax seven months after sirolimus induction, sirolimus was restarted 1.8 months after SPC, and pneumothorax did not recur 58.4 months after SPC. Another patient experienced pneumothorax during cessation of sirolimus and sirolimus was restarted 3.3 months after initial SPC. For the rest of the surgeries in the sirolimus group, sirolimus was induced after SPC. In the non-sirolimus group, only one patient who underwent bilateral SPC started 2 mg of sirolimus after the relapse of pneumothorax bilaterally. The patient experienced another pneumothorax 0.6 months after sirolimus induction; a second SPC was performed, and no further recurrence was observed even 5 months after restarting sirolimus.

## Discussion

The five-year recurrence-free rate was 82.9% in our study. A previous study in 2006 by Almoosa et al. included a surgical procedure for pneumothorax in LAM, and the overall recurrence-free rate was 68% after the surgical procedure [3]; this study lacked detailed information about LAM severity and treatment; thus, the differences in the study population and treatment could not be evaluated accurately. However, over half of the patients in this study experienced two or more episodes of pneumothorax before the SPC; such a high recurrence-free rate might have been influenced by the treatment rather than the study population. Recently, Suzuki et al. have reported that the probability of a 5-year recurrence-free rate of pneumothorax in LAM after TPC was 64.1% [26]. They compared the RFS of mTOR inhibitors (sirolimus, everolimus) initiated patients and RFS of mTOR inhibitors free patients, however there was no significant difference (*p* = 0.723). We suspected that the disease severity could have been different because fewer patients were treated with mTOR inhibitor in their study, despite the lack of detailed clinical data. Their observation period was shorter (median 27 months) than ours, and some cases were excluded from the analysis for mTOR inhibitor treated patients. There have been two reports of preventive effects of sirolimus against recurrence of pneumothorax; these are a case report of an infant with TSC-LAM by Takia et al. [12] and a case series of five patients developing sporadic LAM by Zhoh et al. [13]. The aforementioned reports have concluded that administration of sirolimus reduces the onset of pneumothorax. In our study, the patients started sirolimus at different time points after SPC, and relapse itself might affect the initiation of sirolimus due to side effect of sirolimus of delaying wound healing (only one patient in the non-sirolimus group started sirolimus). However, the fact that SPC with sirolimus induction within a year (n = 6; 37.5%) were also free from postoperative recurrences may contribute to highlighting the efficacy of sirolimus in avoiding recurrent pneumothorax.

An mTOR inhibitor, sirolimus, leads to loss-of-function mutations in TSC genes, which result in the constitutive activation of the mTOR signaling pathway, leading to inappropriate LAM cell growth, invasion, migration, lymphangiogenesis, and destructive tissue remodeling [1] by blocking mTOR activation of downstream kinases and restores homeostasis in cells with defective TSC gene function [27]. Because of this mechanism, sirolimus was reported to stabilize lung function [8] and slow down the increase in cystic lesions in LAM [28]. Therefore, it was assumed that the effect of sirolimus on the reduction of the growth of LAM cells in the lung might affect the incidence of pneumothorax. If sirolimus prevents the onset of pneumothorax, physicians can avoid invasive pleural procedures in LAM. However, physicians should be careful in starting sirolimus because it can delay tissue remodeling, and sirolimus can cause pneumothorax [9]. Therefore, the timing of starting sirolimus after surgery needs to be studied further.

Since four patients required oxygen therapy after SPC in this study, it is possible that SPC is invasive for patients with a low-respiratory function. Of these, three patients could tolerate oxygen therapy withdrawal, and the other patient, who could not sustain oxygen therapy withdrawal, died of progressive respiratory failure 170 days after SPC. The patient had a low percentage of forced expiratory volume (48.7% predicted) and high VEGF-D levels (3624 pg/mL) before SPC. Both thoracotomy and VATS are known to reduce most ventilatory parameters postoperatively, with a precipitous drop occurring by the first operative day [29]. Moreover, TPC is based on the phenomenon that an ORC mesh turns acidic in the human body and induces inflammation of the visceral pleura histologically [4, 7]. SPC is considered to decrease lung compliance temporally. However, if the decrease in lung function after SPC is temporal, SPC is possibly not as invasive as pleurodesis because of lesser adhesion. On TPC, Kurihara reported the postoperative relapse after TPC depended on the number of ORC mesh used during surgery. On the other hand, since two types of reinforce materials were used in this study, we could not compare our results with the aforementioned study accurately. Kim reported one case of TSC-LAM wherein lung transplantation was performed on the same side as TPC; the patient was protected from severe thoracic adhesion after TPC [30]. Therefore, SPC in which ORC is mainly used may be one of the treatment options, especially for patients who need to undergo lung transplantation eventually.

We determined clinical parameters (e.g., high serum VEGF-D level, high %LAV, premenopausal status, oxygen therapy requirement, bronchodilator use). However, none of these factors could predict the risk of postoperative recurrent pneumothorax after SPC and the only predictor of postoperative recurrence was sirolimus treatment after SPC. Although high serum VEGF-D level (> 938 pg/mL) was not predictive for postoperative recurrence by log-rank test, serum VEGF-D was significantly higher in the recurrence group than the no recurrence group. Future studies are required to confirm the relationship between VEGF-D and the development of pneumothorax in LAM. During the perioperative SPC period, no significant difference was observed between the sirolimus and non-sirolimus groups regarding clinical characteristics. Therefore, our results suggest that the severity of LAM did not directly affect the initiation of sirolimus after SPC in our cohort.

### Limitations of this study

Several limitations were inherent. Firstly, this was a retrospective study conducted at a single institution. Due to the rarity of LAM, such a limited population might have led to the underestimation of some factors predicting postoperative pneumothorax recurrence. Secondly, this study could not show the number of reinforce materials using during surgery due to lack of these data in some surgical reports, and there was discussion whether total (entire) lungs were really covered or not. Our pulmonologists and surgeons understood the role of covering entire lung for the treatment of intractable pneumothorax in LAM, and we covered entire pleura according to TPC [5]. In addition, we applied modification with PGA in some cases with air-leak points according to the procedure of mTPC which Noda et al. reportd [6] (Additional file 3). Thirdly, because of pneumothorax itself, the preoperative pulmonary function was lacking, and we could not evaluate the severity of LAM correctly by pulmonary function tests. However, we evaluated it using %LAV, which quantitated the cystic lesion of the lung. There was no significant difference of %LAV between the cases treated by sirolimus and untreated cases. In order to understand the inhibitory effect of sirolimus on postoperative recurrent pneumothorax, a further larger study is required.

## Conclusion

We first reported sirolimus might effectively suppress the recurrence of pneumothoraces in LAM patients who received SPC. Sirolimus induction after SPC (TPC or mTPC) might be a feasible option for frequent pneumothorax in LAM.

## Supplementary Information

**Additional file 1:** The Clavien-Dingo classification of surgical complications [23]. The table is showing the Clavien-Dingo classification which is using for evaluation of surgical complications.

**Additional file 2:** Kaplan–Meier curve showing relapse of pneumothorax as an outcome after initial surgical pleural covering of entire lung including total pleural covering and modified total pleural covering on each clinical parameter. Log-rank test analysis on age (A) (*p* = 0.19), body mass index (B) (*p* = 0.29), existence of angiomyolipoma (C) (*p* = 0.63), percentage of low attenuation volume (D) (*p* = 0.29), episodes of ipsilateral pneumothorax before SPC (E) (*p* = 0.78), use of oxygen therapy (F) (*p* = 0.58), and the use of bronchodilators (G) (*p* = 0.32). In age, body mass index, pneumothorax occurrences, and percentage of low attenuation volume, the groups are divided according to their median value, and none of the factors shows a relationship with postoperative recurrence.

**Additional file 3:** Clinical data of surgical pleural covering of entire lung (SPC, n = 24) including total pleural covering (TPC, with ORC, n = 17) and modified total pleural covering (mTPC, with OCR + PGA, n = 7) in 18 patients with LAM about individual SPC and sirolimus initiation. The table is showing data about SPC and sirolimus initiation individually.

## Data Availability

The datasets used and/or analyzed during the current study are available from the corresponding author on reasonable request.

## Abbreviations

LAM: Lymphangioleiomyomatosis
TSC: Tuberous sclerosis complex
TPC: Total pleural covering
mTPC: Modified total pleural covering
SPC: Surgical pleural covering
ORC: Oxidized regenerated cellulose
PGA: Polyglycolic acid
mTOR: Mechanistic target of rapamycin
VEGF-D: Vascular endothelial growth factor D
KCCMC: National Hospital Organization Kinki-Chuo Chest Medical Center
CT: Computed tomography
BMI: Body mass index
LAA: Low attenuation area
%LAV: Percentage of low attenuation volume
VATS: Video-assisted thoracoscopic surgery
PGA: Polyglycolic acid
PAL: Persistent air leakage
AML: Angiomyolipoma
GnRH: Gonadotropin-releasing hormone
KCCMC: Kinki-Chuo Chest Medical Center
