# Supplementary material for: Reduced risk of recurrent pneumothorax for sirolimus therapy after surgical pleural covering of entire lung in lymphangioleiomyomatosis
Source: Orphanet J Rare Dis. 2021 Nov 3;16:466. doi: 10.1186/s13023-021-02081-z (PMC8567719; doi:10.1186/s13023-021-02081-z)
Supplement: Supplementary file 1 — Additional file 1: The Clavien-Dingo classification of surgical complications [23]. The table is showing the Clavien-Dingo classification which is using for evaluation of surgical complications. [file 13023_2021_2081_MOESM1_ESM.docx]

Additional File 1: The Clavien-Dingo classification of surgical complications (23)

| Grade | Definition |
| --- | --- |
| Grade I | Any deviation from the normal postoperative course without the need for pharmacological treatment or surgical, endoscopic, and radiological interventions |
|  | Allowed therapeutic regimens are: drugs as antiemetics, antipyretics, analgetics, diuretics, electrolytes, and physiotherapy. This grade also includes wound infections opened at the bedside |
| Grade II | Requiring pharmacological treatment with drugs other than such allowed for grade I complications |
|  | Blood transfusions and total parenteral nutrition are also included |
| Grade III | Requiring surgical, endoscopic or radiological intervention |
| IIIa | Intervention not under general anesthesia |
| IIIb | Intervention under general anesthesia |
| Grade IV | Life-threatening complication (including CNS complications)* requiring IC/ICU management |
| Iva | Single organ dysfunction (including dialysis) |
| IVb | Multiorgan dysfunction |
| Grade V | Death of a patient |
| * Brain hemorrhage, ischemic stroke, subarachnoid bleeding, but excluding transient ischemic attacks. CNS, central nervous system; IC, intermediate care; ICU, intensive care unit. | |
