# Supplementary material for: Reduced risk of recurrent pneumothorax for sirolimus therapy after surgical pleural covering of entire lung in lymphangioleiomyomatosis
Source: Orphanet J Rare Dis. 2021 Nov 3;16:466. doi: 10.1186/s13023-021-02081-z (PMC8567719; doi:10.1186/s13023-021-02081-z)
Supplement: Supplementary file 2 — Additional file 2: Kaplan–Meier curve showing relapse of pneumothorax as an outcome after initial surgical pleural covering of entire lung including total pleural covering and modified total pleural covering on each clinical parameter. Log-rank test analysis on age (A) (p = 0.19), body mass index (B) (p = 0.29), existence of angiomyolipoma (C) (p = 0.63), percentage of low attenuation volume (D) (p = 0.29), episodes of ipsilateral pneumothorax before SPC (E) (p = 0.78), use of oxygen therapy (F) (p = 0.58), and the use of bronchodilators (G) (p = 0.32). In age, body mass index, pneumothorax occurrences, and percentage of low attenuation volume, the groups are divided according to their median value, and none of the factors shows a relationship with postoperative recurrence. [file 13023_2021_2081_MOESM2_ESM.pptx]

## Slide 1
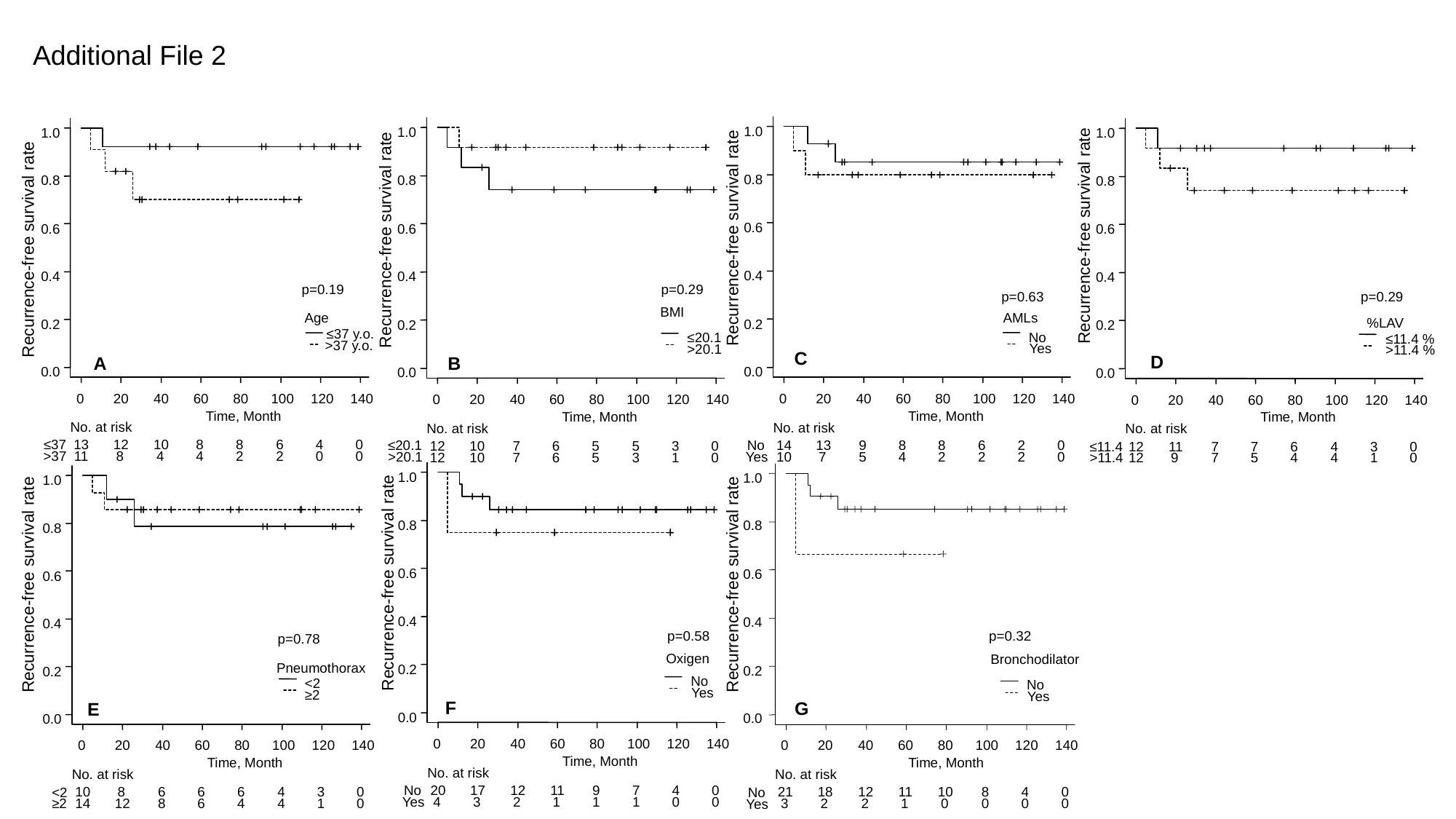

# Additional File 2
1.0
0.8
0.6
Recurrence-free survival rate
0.4
AMLs
0.2
No
Yes
0.0
0
20
40
60
80
100
120
140
Time, Month
No. at risk
14
13
9
8
8
6
2
0
No
10
7
5
4
2
2
2
0
Yes
1.0
0.8
0.6
Recurrence-free survival rate
0.4
BMI
0.2
≤20.1
>20.1
0.0
0
20
40
60
80
100
120
140
Time, Month
No. at risk
≤20.1
12
10
7
6
5
5
3
0
>20.1
12
10
7
6
5
3
1
0
1.0
0.8
0.6
0.4
Age
0.2
≤37 y.o.
>37 y.o.
0.0
0
20
40
60
80
100
120
140
Time, Month
No. at risk
≤37
13
12
10
8
8
6
4
0
>37
11
8
4
4
2
2
0
0
1.0
0.8
0.6
Recurrence-free survival rate
0.4
%LAV
0.2
≤11.4 %
>11.4 %
0.0
0
20
40
60
80
100
120
140
Time, Month
No. at risk
≤11.4
12
11
7
7
6
4
3
0
>11.4
12
9
7
5
4
4
1
0
Recurrence-free survival rate
p=0.19
p=0.29
p=0.63
p=0.29
C
D
B
A
1.0
0.8
0.6
Recurrence-free survival rate
0.4
Oxigen
0.2
No
Yes
0.0
0
20
40
60
80
100
120
140
Time, Month
No. at risk
No
20
17
12
11
9
7
4
0
Yes
4
3
2
1
1
1
0
0
1.0
0.8
0.6
Recurrence-free survival rate
0.4
Bronchodilator
0.2
No
Yes
0.0
0
20
40
60
80
100
120
140
Time, Month
No. at risk
21
18
12
11
10
8
4
0
No
3
2
2
1
0
0
0
0
Yes
1.0
0.8
0.6
Recurrence-free survival rate
0.4
Pneumothorax
0.2
<2
≥2
0.0
0
20
40
60
80
100
120
140
Time, Month
No. at risk
10
8
6
6
6
4
3
0
<2
14
12
8
6
4
4
1
0
≥2
p=0.58
p=0.32
p=0.78
F
G
E
