# Supplementary material for: Reduced risk of recurrent pneumothorax for sirolimus therapy after surgical pleural covering of entire lung in lymphangioleiomyomatosis
Source: Orphanet J Rare Dis. 2021 Nov 3;16:466. doi: 10.1186/s13023-021-02081-z (PMC8567719; doi:10.1186/s13023-021-02081-z)
Supplement: Supplementary file 3 — Additional file 3: Clinical data of surgical pleural covering of entire lung (SPC, n = 24) including total pleural covering (TPC, with ORC, n = 17) and modified total pleural covering (mTPC, with OCR + PGA, n = 7) in 18 patients with LAM about individual SPC and sirolimus initiation. The table is showing data about SPC and sirolimus initiation individually. [file 13023_2021_2081_MOESM3_ESM.docx]

| Case | Laterality | Age | Reinforce Materials | Sirolimus intake before SPC | Pneumothorax before SPC (times) | Recurrence after SPC(month) | Follow-up period from SPC (month) | from SPC to Sirolimus Initiation (month) | Duration of Sirolimus administration (month) |
| --- | --- | --- | --- | --- | --- | --- | --- | --- | --- |
| 1 | R | 36 | ORC | No | 5 | No recurrence | 138.6 | 46.0 | 92.6 |
|  | L | 37 | ORC | No | 1 | No recurrence | 126.9 | 34.3 | 92.6 |
| 2 | R | 23 | ORC+PGA | No | 1 | No recurrence | 125.3 | 98.5 | 26.8 |
|  | L | 30 | ORC+PGA | No | 7 | No recurrence | 37.2 | 10.4 | 26.8 |
| 3 | R | 37 | ORC | No | 2 | No recurrence | 109.7 | - | - |
| 4 | R | 38 | ORC | No | 2 | No recurrence | 109.1 | 42.2 | 66.9 |
| 5 | R | 47 | ORC+PGA | No | 1 | No recurrence | 101.7 | 9.9 | 91.8 |
| 6 | R | 36 | ORC+PGA | No | 1 | No recurrence | 90.3 | 70.3 | 20.0 |
|  | L | 36 | ORC+PGA | No | 1 | No recurrence | 92.5 | 72.5 | 20.0 |
| 7 | R | 47 | ORC | No | 6 | No recurrence | 78.3 | 73.7 | 4.5 |
| 8 | L | 46 | ORC | No | 2 | No recurrence | 74.0 | - | - |
| 9 | L | 43 | ORC | No | 2 | Recurrence (4.9) | 5.7 | - | - |
| 10 | R | 39 | ORC | No | 1 | Recurrence (12.0) | 64.0 | 48.3 | 5.0 |
|  | L | 40 | ORC | No | 1 | Recurrence (25.8) | 50.8 | 35.1 | 5.0 |
| 11 | R | 32 | ORC | No | 2 | No recurrence | 58.4 | 1.8 | 51.6 |
| 12 | R | 37 | ORC | No | 2 | No recurrence | 44.2 | 35.4 | 8.7 |
|  | L | 38 | ORC | No | 2 | No recurrence | 29.2 | 20.5 | 8.7 |
| 13 | R | 26 | ORC | No | 1 | No recurrence | 34.2 | - | - |
|  | L | 26 | ORC | No | 7 | Recurrence (10.9) | 34.2 | - | - |
| 14 | R | 40 | ORC | Yes | 2 | No recurrence | 30.4 | 4.6 | 25.8 |
| 15 | L | 44 | ORC | No | 3 | No recurrence | 22.1 | - | - |
| 16 | L | 37 | ORC+PGA | Yes | 6 | No recurrence | 116.6 | 3.3 | 125.0 |
| 17 | R | 29 | ORC+PGA | No | 1 | No recurrence | 134.6 | 43.0 | 42.9 |
| 18 | L | 47 | ORC | No | 1 | No recurrence | 17.1 | 4.7 | 12.4 |
| R: Right, L: Left, ORC: Oxidized Regenerated Cellulose, PGA: Polyglycolic Acid, SPC: Surgical Pleural Covering | | | | | | | | | |

Additional File 3:
